# Parametric and non-parametric gradient matching for network inference: a comparison

**DOI:** 10.1186/s12859-018-2590-7

**Published:** 2019-01-25

**Authors:** Leander Dony, Fei He, Michael P. H. Stumpf

**Affiliations:** 1 Centre for Integrative Systems Biology and Bioinformatics, Department of Life Sciences, Imperial College London, London, SW7 2AZ UK; 2 Institute of Computational Biology, Helmholtz Center Munich, German Research Center for Environmental Health, Neuherberg, 85764 Germany; 3 Max Planck Institute of Psychiatry, Kraepelinstr. 2-10, Munich, 80804 Germany; 4 School of Computing, Electronics, and Mathematics, Coventry University, Coventry, CV1 2JH UK; 5 Melbourne Integrative Genomics, School of BioScience & School of Mathematics and Statistics, University of Melbourne, Parkville Melbourne, 3010 Australia

**Keywords:** Systems biology, Gradient matching, Gene regulation, Network inference

## Abstract

**Background:**

Reverse engineering of gene regulatory networks from time series gene-expression data is a challenging problem, not only because of the vast sets of candidate interactions but also due to the stochastic nature of gene expression. We limit our analysis to nonlinear differential equation based inference methods. In order to avoid the computational cost of large-scale simulations, a two-step Gaussian process interpolation based gradient matching approach has been proposed to solve differential equations approximately.

**Results:**

We apply a gradient matching inference approach to a large number of candidate models, including parametric differential equations or their corresponding non-parametric representations, we evaluate the network inference performance under various settings for different inference objectives. We use model averaging, based on the Bayesian Information Criterion (BIC), to combine the different inferences. The performance of different inference approaches is evaluated using area under the precision-recall curves.

**Conclusions:**

We found that parametric methods can provide comparable, and often improved inference compared to non-parametric methods; the latter, however, require no kinetic information and are computationally more efficient.

**Electronic supplementary material:**

The online version of this article (10.1186/s12859-018-2590-7) contains supplementary material, which is available to authorized users.

## Background

Gene expression is known to be subject to sophisticated and fine-grained regulation. Besides underlying the developmental processes and morphogenesis of every multicellular organism, gene regulation represents an integral component of cellular operation by allowing for adaptation to new environments through protein expression on demand [1–4].

**Fig. 1:**
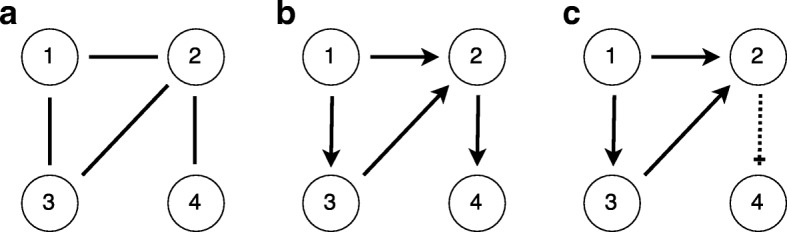
Gene regulatory network (GRN) schematics with four genes and four interactions. Three representations of the same GRN are shown. **a** Undirected graph showing interactions between genes: 1, 2; 1, 3; 2, 3; 2, 4. **b** Directed graph showing interactions between genes (parent node stated first): 1, 2; 1, 3; 3, 2; 2, 4. **c** Directed graph showing interactions between genes: 1 activates 2; 1 activates 3; 3 activates 2; 2 represses 4

While the basic principles of gene regulation have been discovered as early as 1961 [5], understanding the structure and dynamics of complex gene regulatory networks (GRN) remains an open challenge. Gene regulatory interactions within a group of genes can be visualised in various ways. Usually, genes and their interactions are represented as nodes and edges of a graph respectively. Depending on the aim of the study and the employed method, the graph can be undirected (Fig. 1a); directed (Fig. 1b); or contain further information about interaction types (Fig. 1c). With the development of high-throughput expression measurement techniques, there is a rich and growing literature on network reconstruction or inference, ranging from data-driven methods (e.g. correlation-based methods, regression analysis, information theoretical approaches), to probabilistic models (e.g. Gaussian graphical models, (dynamic) Bayesian networks) and mechanistic model-based methods (e.g. Petri nets, Boolean networks, differential equations) [1, 6–12].

Given the vast range of network inference approaches studied within and outside the life sciences, we limit our analysis in this work to infer gene regulatory interactions from time-course data (e.g. time-resolved mRNA concentration measurements) under a nonlinear dynamic systems framework, since most of data-driven methods either purely study the linear interactions or ignore the dynamic information from the data. More specifically, we will investigate the inference based on nonlinear ordinary differential equations (ODEs) and corresponding non-parametric representations.

The application of ODE models in this context has the advantage that each individual term in the final ODE model can provide direct mechanistic insight (such as presence of activation or repression) [13, 14]. Following [13, 15], we employ a general ODE representation of a GRN,

1 $$  \begin{aligned} \dot{x}_{n}(t) &= s_{n} + \beta_{n} \cdot f_{n}(\mathbf{x}(t),\boldsymbol{\theta}_{n},t) - \gamma_{n} \cdot x_{n}(t) \\ &= f(\mathbf{x}(t),\boldsymbol{\alpha}_{n},t). \end{aligned}  $$

Here, *x*_*n*_(*t*) denotes the concentration of *n*^*th*^ mRNA at time *t*, *s*_*n*_ is the basal transcription rate, *γ*_*n*_ is the mRNA decay rate, **x** is a vector of concentrations of all the parent mRNAs that regulate the *n*^*th*^ mRNA, the regulation function *f*_*n*_ describes the regulatory interactions among genes such as activation or repression that are normally quantified by Hill kinetics, with *β*_*n*_ the strength or sensitivity of gene regulation, and the parameter vector ***θ***_*n*_ contains regulatory kinetic parameters. The right-hand-side of the *n*^*th*^ ODE can be summarized in a single nonlinear function *f* with ***α***_*n*_ including all the kinetic parameters. Some approaches such as non-parametric Bayesian inference methods provide less mechanistic information but they may nevertheless provide realistic representations of complex regulatory interactions between genes, which a simple ODE system might not be able to capture [16], especially when accurate kinetic information is unavailable.

Parameter and structure inference of a mathematical model expressed as coupled ODEs (Eq. (1)) is a challenging problem, as repeatedly solving the ODEs by numerical integration is required which is computationally costly. Such costs quickly increase as the number of genes in the network increases. A two-step gradient matching approach has been proposed in the machine learning literature [17–19] to reduce the computational cost: in the first step, the time series data are interpolated, and in the second step, the parameters of ODEs are optimized by minimizing the difference between interpolated derivatives and the right-hand-side of ODEs. Thus the ODEs do not need to be solved explicitly. As the gradients can be sensitive to noise, instead of approximating the derivatives, one can also use integrals by numerical integrating the right-hand-side of the ODEs and minimize its difference with interpolated state trajectories. However, due to the numerical complexity of integrating nonlinear functions practically its applications are limited to ODEs with certain structure, e.g. linear in the parameters [20, 21].

More recently, an improved inference scheme, adaptive gradient matching, has been proposed [22, 23] where GP interpolation is regulated by the ODE system through joint inference of GP hyperparameters and ODE parameters. This way an improvement on the robustness of parameter inference with respect to noise can be achieved. In the network inference context, however, due to a large number of candidate models which need to be inferred and the corresponding computational cost, we will not evaluate this adaptive scheme explicitly in this work.

Previous work in the field of automatic network reconstruction has proposed a gradient matching approach to triaging different network topologies [13, 24]. Gradient matching for automatic ODE network reconstruction combined with Gaussian process (GP) regression could be a promising avenue for inferring GRNs. But still, some problems remain: model identifiability, as too many models provide a good fit to the data; reliably fitting GPs to noisy data; and potentially limiting model assumptions, e.g. by considering only a limited range of interaction types.

**Fig. 2:**
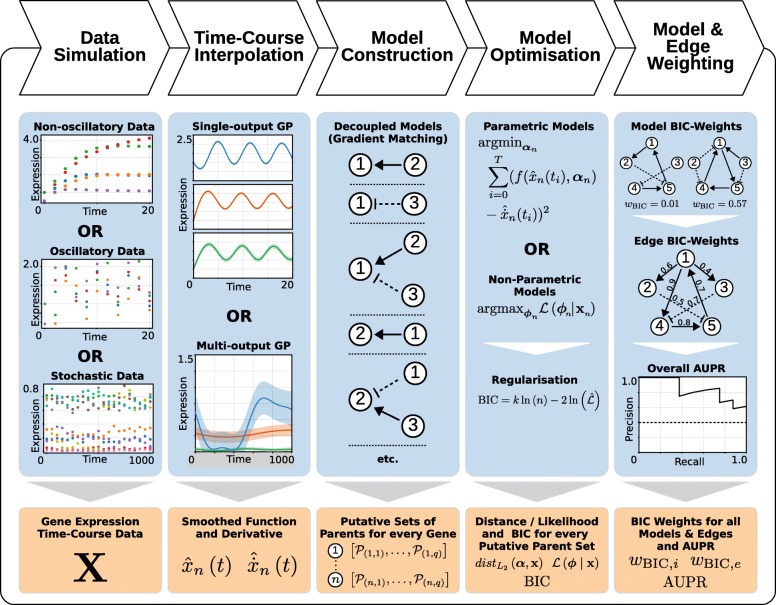
Pipeline outline schematic. This figure illustrates the five main steps in the network inference pipeline developed in this project. Mathematical symbols and expressions used in this figure are defined and explained in the relevant sections of the main text. All numbers and schematics are shown purely for illustration and do not reflect actual results. Abbreviations used here are: **GP** – Gaussian Process; **BIC** – Bayesian Information Criterion; **AUPR** – Area under the precision-recall curve

In this work, we investigate and attempt to address those issues and furthermore evaluate inference performance of gradient matching approach under different conditions. We structure our work by comparing the inference performance of parametric and non-parametric inference methods as described in Fig. 2.

## Methods

This section outlines the different approaches taken to reconstruct GRN. Details on the software and algorithms employed can be found in Additional file 1, Section 4.

### Gene expression data

To compare different network inference approaches and settings, we simulate deterministic gene expression data from a relative small 5-gene regulatory network. We then repeat the analysis using more realistic stochastically simulated data generated from a 10-gene regulatory network in *Saccharomyces cerevisiae*.

#### Deterministic ODE model simulation

**Fig. 3:**
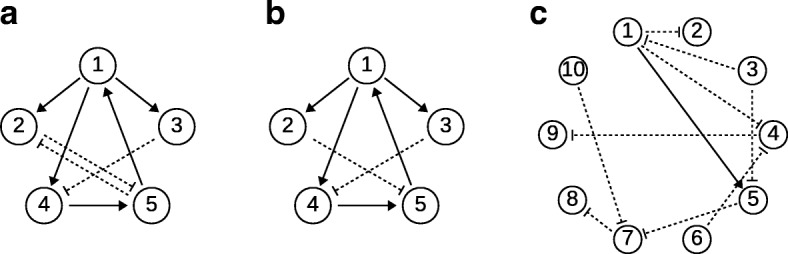
Schematics of gene regulatory networks used in this work. **a** Five-gene network with eight interactions used to simulate the ‘non-oscillatory noise-free data’. **b** Five-gene network with seven interactions used to simulate the ‘oscillatory noise-free data’. **c** Ten-gene network with ten interactions used by GeneNetWeaver to simulate the ‘realistic’ stochastic expression data

We use deterministically simulated gene expression data based on the *in vivo*benchmarking of reverse-engineering and modelling approaches (IRMA) network [25]. The IRMA network is a quasi-isolated synthetic five-gene network, constructed in *Saccharomyces cerevisiae* (Fig. 3a). We refer to this dataset as ‘non-oscillatory data’.

To ensure comparability to previous work with this model [13, 24], we use the same model parameters and also create a second subset with one edge removed (Fig. 3b) and regulatory interactions modelled as previously [13, 24, 26]. We refer to this dataset as ‘oscillatory data’. For completeness we provide the structure of the ODE systems as well as the parameters and settings used for simulation once again in Additional file 1, Section 1.1.

#### Simulated stochastic gene expression data

In order to evaluate the performance of different inference methods with more realistic stochastically simulated gene expression data (that are not directly generated under our ODE model assumptions), we use GeneNetWaver [27] to generate realistic gene expression profiles from a simulated ten-gene network (Fig. 3c) from *Saccharomyces cerevisiae* (as previously used in the DREAM3 and DREAM5 challenge [28]). The dataset we used is referred to as *InSilicoSize10-Yeast1_dream4* in GeneNetWeaver. We obtain data for the same 20 time points for every gene.

GeneNetWaver [27] simulates realistic noisy gene expression data by introducing process noise (through stochastic differential equations) as well as observational noise to the underlying gene expression profiles.

### Data smoothing with Gaussian processes

For smoothing and interpolation of the potentially noisy gene expression data, we use Gaussian process (GP) regression. This also allows us to obtain the rate of change in the expression via the GP derivative, which is analytically obtainable. In this section we only provide a very brief introduction to the theoretical foundations of GPs and mainly focus on outlining our choices and settings used in the GP framework. For more details, we refer to [29–31].

#### Gaussian process regression

A GP is defined by a mean *m* and covariance function *k*, so that we can write $f(t) \sim \mathcal {GP}(m,k)$ for any suitable function *f*. Any finite collection of values from *f*(*t*) are hence distributed according to a multivariate Gaussian distribution and so we can write $\left [f(t_{1}),\ldots,f(t_{D})\right ] \sim \mathcal {N}\left (\mathbf {m},K\right)$. **m** describes the vector of *D* mean values and $K = k\left (t,t^{\prime }\right)$ is the covariance matrix, where the value of each element is defined by the GP covariance function.

We use a zero mean function and employ the common squared exponential covariance function [29], which defines the covariance between two observations at time points *t* and *t*^′^ as,

2 $$  k\left(t,t^{\prime}\right) = \sigma^{2}_{f} \exp \left(\frac{-\left(t-t^{\prime}\right)^{2}}{2l^{2}}\right),  $$

with $\sigma ^{2}_{f}$ controlling the variance (‘amplitude’) of the the GP, and the length-scale *l* controlling how many data points around the current one are taken into account when fitting the GP.

We optimise the hyperparameters ***ϕ***={*σ*_*f*_,*σ*_*n*_,*l*} by maximising,

3 $$  \begin{aligned} \ln p\left(\mathbf{x}\mid \mathbf{t}, \boldsymbol{\phi}\right) = &-\frac{1}{2} \mathbf{x}^{\top} (K+\sigma^{2}_{n}I)^{-1} \mathbf{x} \\ &-\frac{1}{2} \ln |K+\sigma^{2}_{n}I| -\frac{D}{2}\ln 2\pi, \end{aligned}  $$

where $\sigma ^{2}_{n}$ denotes the variance of the observational noise and we can write $x(t) \sim \mathcal {N}\left (f(t), \sigma ^{2}_{n}\right)$, *K* corresponds to the covariance matrix and *D* denotes the number of observations in vector **x**. **t**, **x**∈**R**^*D*^.

We obtain predictions **x**_∗_ at time points $\mathbf {t}_{*}=\left [t_{1}^{*},t_{2}^{*},\ldots,t_{S}^{*}\right ]$ from the GP model, since the joint (prior) probability distribution of the training output **x** and testing output **x**_∗_ is again multivariate Gaussian,

4 $$ \left[\begin{array}{c}\mathbf{x}\\\mathbf{x}_{*}\end{array}\right] \sim \mathcal{N}\left(0, \left[\begin{array}{cc}K+\sigma^{2}_{n}I & K_{*}\\K^{\top}_{*} & K_{**}\end{array}\right]\right),  $$

where $K = k\left (t,t^{\prime }\right)$, $K_{*} = k\left (t,t^{\prime }_{*}\right)$, $K^{\top }_{*} = k\left (t_{*},t^{\prime }\right)$ and $K_{**} = k\left (t_{*},t_{*}^{\prime }\right)$.

The posterior distribution of the output at **t**_∗_ can be calculated as,

5 $$ \mathbf{x}_{*}|\mathbf{x} \sim \mathcal{N} \left(K_{*}\left(K+\sigma^{2}_{n}I\right)^{-1}\mathbf{x}, K_{**}-K_{*}\left(K+\sigma^{2}_{n}I\right)^{-1}K^{\top}_{*}\right).  $$

#### Gaussian process derivatives

We can also directly obtain the derivatives of the GP mean values, representing the rate of change in mRNA concentration $\dot {\mathbf {x}}_{*}$, as the derivative of a GP is again a GP [30, 32],

6 $$ \begin{aligned} {\frac{d\mathbf{x}_{*}}{dt}} &= L_{*}\left(K+\sigma^{2}_{n}I\right)^{-1}\mathbf{x},\\ \left[L_{*}\right]_{ij} &= \frac{d}{dt_{j}^{*}} k\left(t_{i},t_{j}^{*}\right)=\frac{\left(t_{i}-t_{j}^{*}\right)}{l^{2}}\left[K_{*}\right]_{ij}. \end{aligned}  $$

The derivatives obtained here will also be used for the gradient matching inference algorithm to be discussed next.

#### Multiple output gaussian processes

Standard GP regression allows us to make predictions on the expression level of a single gene. To improve the GP fitting to multiple genes, intrinsic coregionalisation for multi-output GP regression [33] ia employed. This is a form of a multiple output GP [34] which takes into account correlation between the expression of all genes in the network through a correlated noise process. Considering a system with *N* outputs, the overall covariance (or kernel) matrix *K* of the multi-output GP takes the form,

7 $$ K\left(X,X\right) = B \otimes k\left(X,X\right),  $$

where *B*∈**R**^*N*×*N*^ is the coregionalisation matrix, ${X=\{\mathbf {x}_{i}\}_{i=1}^{N} \in \mathbf {R}^{ND}}$ is the input vector that contains observations for all the *N* outputs, and ⊗ denotes the Kronecker product. If *B*=*I*_*N*_, then all outputs are uncorrelated. The hyperparameters in the covariance function $k\left (X,X\right)$ and *B* can be estimated jointly via the eigen-decomposition of the matrix *B* and maximum likelihood estimation [35].

We obtain the smoothed mRNA concentration values from the mean function of the GP. Since computing the derivatives of a multi-output GP is relatively complicated, we approximate the derivative at each point numerically,

8 $$ \frac{dx}{dt} \approx \frac{x\left(t+\delta\right)-x(t)}{\delta}.  $$

Here we use *δ*=10^−4^ as a trade-off between the approximation accuracy and the sensitivity to the noise.

### Model construction and optimisation through gradient matching

We use a gradient-matching parameter optimisation approach to evaluate the goodness of fit of our model to the data [13, 16, 24]. Instead of solving the ODE systems, we directly compute the gradient of the gene expression data using GP regression and then optimise the parameters of the ODE system.

As gradient matching can be carried out for each equation of the ODE system independently, the number of possible network topologies we have to consider reduces drastically. For the five gene network (*N*=5) with two alternative interaction types (*F*=2) and no self-interactions, we only have to consider $N \cdot \sum ^{N-1}_{i=0} \left (\binom {N-1}{i} \cdot F^{i}\right) = 405$ topologies, given the decoupled system (opposed to 3.5·10^9^ fully coupled models). We can further limit the number of topologies by restricting the number of maximum parents per gene (e.g. the maximum in-degree of every gene in the network). For such a small scale network, we set *M*=2 parents per gene (*M*=3 is also evaluated in the simulation study), which would further reduce the space of candidate topologies to $N \cdot \sum ^{M}_{i=0} \left (\binom {N-1}{i} \cdot F^{i}\right) = 165$.

#### ODE models

As during data simulation we use two different approaches to model activation and repression during network inference. The parameters and constraints used for model optimisation are provided in the Additional file 1, Section 1.2.

For the *n*^*th*^ ODE we minimize the *L*_2_ (squared) distance between the constructed parametric function $f(\hat {x}_{n}(t),\boldsymbol {\alpha }_{n})$ (with parameter vector ***α***_*n*_) and the associated derivative calculated from the GP regression $\hat {\dot {x}}_{n}(t)$ for all *S* time points [ *t*_0_,…,*t*_*S*_]:

9 $$  \text{dist}_{L_{2},n} = \sum\limits_{i=0}^{S} \left(f(\hat{x}_{n}(t_{i}),\boldsymbol{\alpha}_{n}) - \hat{\dot{x}}_{n}(t_{i})\right)^{2}.  $$

#### Non-parametric models

We also consider a fully non-parametric, GP-based gradient matching inference method adapted from [16]. This is particularly useful when the detailed reaction kinetics (i.e. ODEs) are unknown and when we are more interested to infer the network interactions instead of the kinetics or reaction types (i.e. activation or repression). Similar to the decoupled ODE system described in the previous section, the gradient matching approach can also be integrated with non-parametric GP regression. This allows for treating each gene *n* conditionally independent of all other genes given its parents $\mathcal {P}_{n}$. We model each gene using the relationship:

10 $$  \dot{x}_{n}(t) = f(\{\mathbf{x}_{q}(t)\mid q \in \mathcal{P}_{n}\},\boldsymbol{\phi}_{n}),  $$

where $f(\{\mathbf {x}_{q}(t)\mid q \in \mathcal {P}_{n}\},\boldsymbol {\phi }_{n}) \sim \mathcal {GP}(0,k)$ is a single-output-multiple-input GP with ***ϕ***_*n*_ denoting the vector of hyper-parameters for the squared exponential covariance function $k \left (t,t'\right)$ (Eq. (2)) for gene *n*. The derivative of the *n*^*th*^ gene expression $\dot {x}_{n}(t)$ can again be obtained from the derivative GP process. Optimisation of each putative GP model is via optimising the hyper-parameters of the covariance function by maximizing the likelihood function.

As this is a purely data-driven approach, basal transcription and degradation are not treated separately as in the ODE approach. Because the degradation of mRNA is usually modelled as a first order reaction, we include gene self-interaction in every putative network. This does not affect the total number of candidate topologies. Furthermore, as this approach is unable to distinguish alternative regulatory types (activation or repression) between genes so that the number of possible network topologies is reduced to $N \cdot \sum ^{M}_{i=0} \binom {N-1}{i} = 55$ (with *M*=2 and *N*=5). Symbol definitions as previously stated in this section.

### Model selection and edge weighting

Following model optimisation, we obtain the final distance or likelihood of each gene with respect to their possible parents which we can use to calculate the Bayesian information criterion (BIC) for each model. For the ODE-based inference approach we have,

11 $$  \text{BIC} = \ln(S) \cdot G + S \cdot \ln\left(\frac{\text{dist}_{L_{2}}}{S}\right),  $$

where *S* denotes the number of data points (sample size), *G* the number of free parameters and $dist_{L_{2}}$ the *L*_2_ distance defined in Eq. (9). Alternatively, for the non-parametric inference approach we obtain,

12 $$  \text{BIC} = \ln(S) \cdot G - 2 \cdot \ln\left(\mathcal{L}\left(\boldsymbol{\hat{\phi}}_{MLE}\mid \mathbf{x}\right)\right).  $$

*S* and *G* are defined as before and $\mathcal {L}\left (\boldsymbol {\hat {\phi }}_{MLE}\mid \mathbf {x}\right)$ denotes the maximum likelihood of the model with optimised hyperparameters $\boldsymbol {\hat {\phi }}_{MLE}$ given gene expression data **x**. We use the BIC for weighting candidate models rather than the commonly used Akaike information criterion (AIC), as it is asymptotically valid for large sample sizes [36] whereas AIC tends to prefer overly complicated models in this case.

We then calculate the Schwarz weight [37] for each model $w_{i}\left (\text {BIC}\right)$ in the set of models *j*,

13 $$ w_{i}\left(\text{BIC}\right) = \frac{\exp\left(\frac{-\Delta_{i}\left(\text{BIC}\right)}{2}\right)}{{\sum\nolimits}_{j} \exp\left(\frac{-\Delta_{j}\left(\text{BIC}\right)}{2}\right)},  $$

such that ${\sum \nolimits }_{i} w_{i}\left (\text {BIC}\right) = 1$. $\Delta _{i}\left (\text {BIC}\right) = \text {BIC}_{i} - \text {BIC}_{min}$ denotes the difference between the BIC of model *i* (BIC_*i*_) and the lowest BIC across all models considered (BIC_*min*_).

Once we have weighted all models across all genes in the network, we can calculate the weight *w*_*e*_ associated with every edge *e* in the GRN. This is done for each edge by summing the Schwarz weight of every model that contains the edge in question,

14 $$ w_{e} = \sum\limits_{i} w_{i}I_{e}(i),  $$

where *I*_*e*_(*i*) denotes the indicator function which is 1 if edge *e* is present in model *i* and 0 otherwise.

### Performance evaluation

To evaluate the overall performance of the GRN inference, we use the BIC weights of every edge in the network to calculate the Area Under the Precision-Recall (AUPR) curve [38]. The detailed explanations and definitions of this AUPR approach are provided in Additional file 1, Section 1.3.

Considering the sparsity of large GRNs, we use the AUPR instead of the Area Under the Receiver Operating Characteristic (AUROC) curve [39] to evaluate performance.

## Results

### Deterministically simulated gene expression data

**Fig. 4:**
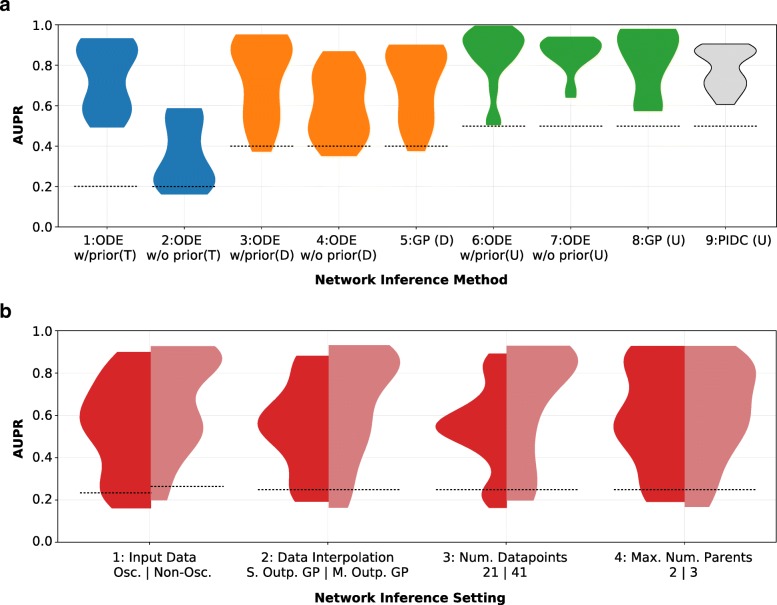
Performance comparison of network inference approaches using noise-free data. **a** This subfigure displays the distribution of obtained performance (AUPR) for the three different classes of network inference methods, over all model settings listed in Table 1. There are four different network inference aims shown in four different shades. The blue distributions relate to the performance of the ODE methods with and without prior at inferring a directed GRN including information about interaction types (activation/repression) **(T)**. The orange distributions depict the performance of the two ODE-based methods and the GP-based method at predicting a directed GRN without type information **(D)**. The green distributions show the performance of the same three methods at inferring an undirected GRN **(U)**. The performance of a recently developed algorithm [10] based on partial information decomposition for the same settings and data is shown as the last distribution in grey (“PIDC”). **b** This subfigure shows the impact of different settings choices on network inference performance. Summing the two halves of each of the four asymmetric distributions in the figure gives rise to the same distribution of model performance (constituted by the three approaches discussed earlier, i.e. the sum of distributions 1, 2 and 5 in Fig. 4A). The dashed line represents baseline (random) performance in all charts

**Fig. 5:**
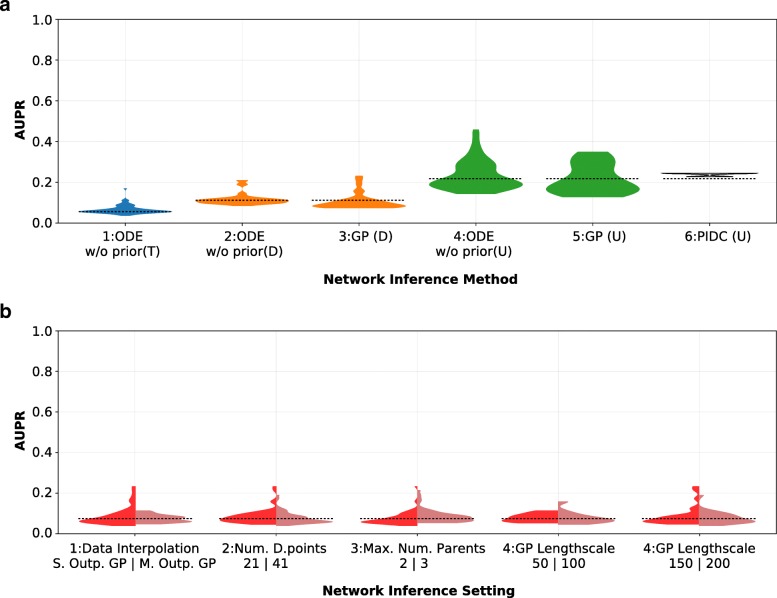
Performance comparison of network inference approaches using realistic simulated data. **a** This subfigure displays the distribution of obtained performance (AUPR) for the three different classes of network inference methods, over all model settings listed in Table 1. There are four different network inference aims shown in four different shades. The blue distribution relates to the performance of the ODE method without prior at inferring a directed GRN including information about interaction types (activation/repression) **(T)**. The orange distributions depict the performance of the ODE-based method and the GP-based method at predicting a directed GRN without type information **(D)**. The green distributions show the performance of the same three methods at inferring an undirected GRN **(U)**. The performance of a recently developed algorithm [10] based on partial information decomposition for the same settings and data is shown as the last distribution in grey (“PIDC”). **b** This subfigure shows the impact of different settings choices on network inference performance. Summing the two halves of each of the first three asymmetric distributions in the figure (and the four parts of the distributions labeled “4”) gives rise to the same distribution of model performance (constituted by the two main approaches discussed earlier (GP and ODE) - i.e. the sum of distributions 1 and 5 in Fig. 5a).The dashed line represents baseline (random) performance in all charts

For the deterministically simulated gene expression data, we compare three main approaches to network inference (Table 1: ‘Inference method’). All three methods are combined with gradient matching. For each inference approach, we evaluate a range of different settings (Table 1) using the AUPR. For the detailed model and parameter settings, please see Additional file 1, Sections 1.1 and 1.2. We present the results in two separate figures (one for noise-free input data (Fig. 4) and one for realistic stochastic data (Fig. 5). Each of the two figures consists of two subplots. Subplot A compares the inference performance for different network modelling scenarios (ODE, GP etc.). Each (asymmetric) violin in subplot B on the other hand compares inference performance over all approaches for a single parameter change (such as using multiple output GPs instead of single output GPs for smoothing the data). For all charts, the width of the shown distribution at any point refers to the relative number of approaches which achieved this particular performance (AUPR). The higher the AUPR, the better the inference performance.

**Table 1:** Employed settings for different network inference approaches

Parameter	Settings
Inference method	Gaussian Process only
	(non-parametric),
	ODE with prior,
	ODE without prior
Input data	Non-oscillatory data (deterministic,
	5 genes, 8 interactions),
	Oscillatory data (deterministic,
	5 genes, 7 interactions),
	Realistic simulated data (stochastic,
	10 genes, 10 interaction)
Data interpolation	Independent single-output GPs,
	Multiple-output GP
Number of datapoints	21, 41
Max. num. of parents	2, 3
Fixed GP length-scale	50, 100, 150, 200
(realistic data only)	

All data presented in this section represent the mean of five independent repeats. It should be noted that in cases of noisy datasets, the number of repetitions should practically be selected according to the confidence intervals of the dataset.

#### Comparing parametric and non-parametric inference

Figure 4a contrasts the performance the three inference approaches across all settings and for three different inference aims, respectively. Only the parametric ODE-based methods allow for distinction between activating and repressing regulatory interactions between genes. From Fig. 4a, we can however clearly see that this type of inference is successful only if the detailed kinetic information about the GRN is available prior to inference: the ODE-based modelling without prior of interactions shows a significant drop in performance over the tested settings compared to the approach with prior where basal transcription and degradation rates are known and ODE parameter ranges can be constrained a priori (see Additional file 1, Section 1.2, Table S1 for parameters).

If we are only interested in the directionality of interactions and not their specific type, the three orange distributions in Fig. 4a show that constraining the parameters of the ODE-based approach (and assuming known basal transcription and degradation rate) is no longer important for achieving good inference performance. The GP-based approach achieves on average higher performance on the simulated datasets used here. This is surprising, since gene interactions used in generating the data are of the same functional form assumed in the ODE inference.

The same trend (with slightly higher overall performance) can be seen when we are only predicting undirected edges. Interestingly, despite higher overall performance, constraining the ODE parameters can lead to worse performance under certain inference settings for this task (compare plot 6 and 7 in Fig. 4a). All three approaches generally perform better on this simple noise-free five-gene networks than the PIDC approach [10].

Below, we analyse the impact of individual factors, i.e. measurement input data type, interpolation method, number of data samples and maximum number of parents, on the overall inference performance of the discussed methods.

#### Input data

The distributions separated by the two input data types (plot 1, Fig. 4b) show a slight performance increase for the non-oscillatory dataset over the oscillatory one. This counterintuitive result can be explained through the increased sensitivity of the GP derivative to imperfect fitting of the oscillatory trajectories compared to the non-oscillatory data which affects the gradient matching based inference result.

This shows that careful consideration has to be placed on both the experimental design step prior to inference (producing data that bears maximum information about the system) [40, 41] as well as on the limiting constraints that the gradient matching approach places on the data (small errors in data fitting due to fluctuations or noise in the data are likely to be amplified in the derivative of the fit).

#### Data interpolation

Despite the deterministic nature of the data we use for evaluation in this section, we find a pronounced difference in performance depending on the method used for interpolating the input data. By taking into account the correlation between the different gene expression time-courses, interpolation with a multiple output GP is able to achieve significantly better results compared to using independent GPs.

When interpolating oscillatory data using single output GPs, we observe that for low number of data points, the GP hyperparameters are optimised so that the oscillatory behaviour is no longer traced by the GP mean, but rather interpreted as noise (Additional file 1, Section 3, Figure S9a). This was also observed in previous work [13]. As shown in Additional file 1, Section 3, Figure S9b this problem can be overcome by using multiple output GP regression, where the oscillatory behaviour correctly traced because trajectories of all genes are taken into account when optimising hyperparameters [42, 43].

#### Number of data points

Plot 3 of Fig. 4b demonstrates increased performance as more time points are used. While this is unsurprising for noise-free data, we will re-evaluate this observation for stochastic data below.

#### Maximum number of parents considered

In Fig. 4b we can see that the maximum number of parents considered per gene does not markedly affect performance. From this we can infer that for noise-free data the regularisation using the BIC efficiently prevents the pipeline from choosing overly complex models. We acknowledge however that computational constraints might require a limitation of of the maximum number of parents in the candidate models.

### Stochastic gene expression data

Gene expression is a stochastic process and we apply the same inference procedures to stochastically simulated gene expression data (but for 10 instead of 5 genes).

#### Comparing parametric and non-parametric inference

The most notable difference between the results for the noise-free and noisy gene expression data is the absolute decline in performance, which is not unexpected. Despite this difference, we nevertheless observe similar trends as for the noise-free data. The ODE-based modelling without prior (plot 2, Fig. 5a) again provides comparable performing result to the non-parametric GP-only modelling approach (plot 3, Fig. 5a) when interaction types are not of interest.

When trying to infer only the existence of (undirected) edges between genes, we observe that the ODE-based model without prior performs slightly better than the GP-based approach; and both approaches perform better than PIDC.

The pronounced narrowing of distributions towards higher AUPR across different approaches indicates that unlike inference based on noise-free data, both ODE and GP-based methods only produce meaningful results (i.e. significantly better than random performance) for a very narrow range of scenarios.

#### Model settings

Contrasting the performance for noise-less and noisy data shows not just lower absolute performance for each method for noisy data, but also different trends of their behaviour (Fig. 5).

Interestingly, we can see from plot 1 of Fig. 5b that in case of stochastic data, all well-performing inference approaches use single output GP interpolation of the data. This could be explained by the large number of free parameters in multiple output GP optimisation. For a ten-gene network, moving from ten independent single output GPs to one 10-output GP means solving a 32-parameter optimisation problem (31 for fixed length-scale) in contrast to solving ten 3-parameter problems. As finding the optimal solution in such a high-dimensional parameter space is extremely difficult, this may be the leading cause for this observation. We further substantiated this by interpolating gene expression data from a smaller GRN using single- and multiple output GP regression and comparing network inference results. An additional reason for the reduced performance could be the limitation to a single length-scale hyperparameter for multiple output GP, while single output GPs can have a different length-scale and variance for every gene they fit. This allows for more flexibility during interpolation. Multiple-output GP methods which allow for varying length-scales are available [44], however, but this further increases the number of free hyperparameters to be optimised.

We also see from plot 2 of Fig. 5b that increasing the number of data points taken from the interpolated data no longer improves performance. While this might seem counter-intuitive at first, the inability of the GP to interpolate the true underlying gene expression dynamics renders the benefit of more data points futile; it appears that GPs can overfit the noise in the data (unless the GP hyperparameters are specifically constrained); using fewer time points can partially compensate for such overfitting. On closer inspection we find that this effect is particularly pronounced for the derivatives obtained from the GPs that play a major role in the inference.

Again changing the maximum number of parents allowed for a gene appears to have no effect (plot 3, Fig. 5b). The rightmost two plots of Fig. 5b show clear evidence for the importance of the right choice of length-scale during data interpolation (only at a length-scale of 150 can an inference performance of AUPR>0.2 be achieved for this example).

## Discussion

In this work, we compare the performance of different network inference methods, especially parametric and non-parametric gradient matching methods, under different settings and scenarios in order to gain an understanding of the strengths, weaknesses and impact of different modelling choices.

When inferring GRNs from limited and inherently noisy gene expression data, there are usually a large number of potential models that can match the data [24]. By computing weights for each model and consequently each interaction in the network, we are able to obtain useful inferences by pooling over different methods.

We find that the simple non-parametric inference approach achieves slightly lower performance than the ODE method without prior despite the absence of mechanistic knowledge about the underlying regulatory processes. It was however shown in previous studies, that a more advanced non-parametric approach which combines Bayesian linear regression and GPs is able to achieve higher performance [16] assuming that some of the parameters are known. In our work, we show that knowledge of such parameters prior to network inference can strongly increase performance and even allows us to infer mechanistic aspects of interactions from data. It is interesting to note, that in particular for the reconstruction of directed GRNs from stochastically simulated gene expression data, inference performance of most methods is not significantly better than random guessing performance. This highlights the difficulty of the GRN inference problem in general.

When inferring networks from gene expression data, the ability of the GP to reconstruct the underlying time-courses from noisy data is a critical factor. Especially the gradient obtained from the GP for the gradient matching procedure is particularly sensitive to poor fits. In order to alleviate this, previous work [13, 23] has suggested employing adaptive gradient matching which can improve performance by taking into account the structure of the ODE model (in case of parametric modelling) during GP fitting. We believe that this approach is still worth pursuing further.

Another promising avenue we see for future work is the combination of parametric and non-parametric methods. A possible approach would be to use the computationally cheaper non-parametric approach to sufficiently narrow the space of possible networks. We could then use ODE-based network inference to confirm interactions as well as obtain mechanistic information for the predicted edges in the GRN. For larger network sizes, this would significantly reduce the computational cost and would therefore make this method suitable to perform inference for network sizes as they are often encountered in experimental studies. If the space of putative networks is small enough following the non-parametric step, we could even avoid decoupling the network which would further increase inference performance.

## Conclusion

In this work, we have carried out a comprehensive comparison of a range of parametric and non-parametric gradient-matching-based approaches on gene regulatory network inference from gene expression data.

We found that applying parametric ODE-based approaches on deterministic gene expression data showed that mechanistic information (such as the type of interaction) can be recovered during inference if enough knowledge about the network (e.g. parameter ranges) is present. For directed and undirected network inference, the parametric ODE method can provide comparable or even better inference performance compared to the non-parametric GP-based method, the latter approach however requires little mechanistic or kinetic regulatory information and computationally more efficient, which can be crucial for large-scale network inference problems. When applied to larger network or stochastic data, overall lower inference performance is observed for all methods, while consistent comparable performance between parametric and non-parametric methods is still obtained.

Several promising avenues to improving inference performance emerge from this analysis: in particular there is potential for the use of multiple output Gaussian Processes for data interpolation in cases of small networks. When applying the same methods to more complex stochastic networks these may, however, become less reliable.

A central result has been that Bayesian model averaging has real potential to increase the quality of network inference. We believe that combining the strengths of several existing approaches will ultimately be required to make significant further progress in solving this challenging problem.

## Additional file

Additional file 1 Contains additional information on some technical aspects of the research. (PDF 340 kb)

## Abbreviations

AIC: Akaike information criterion
AUPR: Area under the precision-recall curve
AUROC: Area under the receiver operating characteristic curve
BIC: Bayesian information criterion
DREAM: Dialogue on reverse engineering assessment and methods
GP: Gaussian process
GRN: Gene regulatory network
IRMA: *In vivo*benchmarking of reverse-engineering and modelling approaches
ODE: Ordinary differential equation
PIDC: Partial information decomposition
