# Supplementary material for: Parametric and non-parametric gradient matching for network inference: a comparison
Source: BMC Bioinformatics. 2019 Jan 25;20:52. doi: 10.1186/s12859-018-2590-7 (PMC6346534; doi:10.1186/s12859-018-2590-7)
Supplement: Supplementary file 1 — Contains additional information on some technical aspects of the research. (PDF 340 kb) [file 12859_2018_2590_MOESM1_ESM.pdf]

Parametric and non-parametric gradient matching for network inference: a comparison

Supplementary Material

Leander Dony[1,2,3], Fei He[1,4], and Michael PH Stumpf[1,5] *

[1]Centre for Integrative Systems Biology and Bioinformatics, Department of Life Sciences, Imperial College London, SW7 2AZ London, UK.

[2]Institute of Computational Biology, Helmholtz Center Munich, German Research Center for Environmental Health, 85764 Neuherberg, Germany.

[3]Max Planck Institute of Psychiatry, Kraepelinstr. 2-10, 80804 Munich, Germany.

[4]School of Computing, Electronics, and Mathematics, Coventry University, CV1 2JH Coventry, UK.

[5]Melbourne Integrative Genomics, School of BioScience & School of Mathematics and Statistics, University of Melbourne, 3010 Parkville Melbourne, Australia

Contents

1 Methodology - Further Details **2**

1.1 Parameters, Functional Forms and Data for ODE Model Simulation 2

1.2 Parameters for Model Optimisation 6

1.3 Performance Evaluation using Precision-Recall curves 7

2 Supplementary Figures for Settings Evaluation **9**

3 Supplementary Figures for fitting GPs **11**

*Corresponding author, email: mstumpf@unimelb.edu.au

4 Software Tools **12**

4.1 Data Simulation 12

4.2 Gaussian Process Regression 12

4.3 Parameter Optimisation 12

1 Methodology - Further Details

1.1 Parameters, Functional Forms and Data for ODE Model Simulation

For simulation of the non-oscillatory data, we model the expression of each gene in the network shown in figure 3A (main text) as ODEs of the form (as used in the DREAM7 (Dialogue on Reverse Engineering Assessment and Methods) challenge [**?**]):

$$\dot{x}_n(t) = s_n - \gamma_n \cdot x_n + \beta_n \cdot f_n^+(\{\mathbf{x}_q(t) \mid q \in \mathcal{P}_n^+\}, \boldsymbol{\theta}_n^+, \mathbf{m}_n^+) \cdot f_n^-(\{\mathbf{x}_q(t) \mid q \in \mathcal{P}_n^-\}, \boldsymbol{\theta}_n^-, \mathbf{m}_n^-) \quad (1)$$

with the following two expressions defining the activating (f_n^+) and repressing (f_n^-) regulation of gene n respectively:

$$f_n^+(\mathbf{x}_n(t), \boldsymbol{\theta}_n^+, \mathbf{m}_n^+) = \sum_{q \in \mathcal{P}_n^+} \left(\frac{x_q(t)^{m_{nq}}}{\theta_{nq}^{m_{nq}} + x_q(t)^{m_{nq}}} \right) \quad (2)$$

$$f_n^-(\mathbf{x}_n(t), \boldsymbol{\theta}_n^-, \mathbf{m}_n^-) = \prod_{q \in \mathcal{P}_n^-} \left(\frac{1}{1 + \left(\frac{x_q(t)}{\theta_{nq}} \right)^{m_{nq}}} \right) \quad (3)$$

Here, $\dot{x}_n(t)$ denotes the rate of change of the mRNA concentration at time t, $x_n(t)$ the mRNA concentration, s_n the basal transcription rate, γ_n the mRNA degradation rate, β_n the strength of gene regulation, all with respect to gene $n \in [1, \dots, i]$ where i denotes the number of genes in the network. $\mathcal{P}_n^+$ and $\mathcal{P}_n^-$ denote the subsets of parent genes which have an activating or repressing effect on the expression of gene n respectively. θ_{nq} and m_{nq} are the commonly used hill parameters for the regulation of gene n by its parent q [**?**].

For simulation of the oscillatory data, we model the expression of each gene in the network shown in figure 3B (main text) as ODEs of the form (parameter notation as in equation 1):

$$\dot{x}_n(t) = s_n - \gamma_n \cdot x_n + \sum_{q \in \mathcal{P}_n} \beta_{nq} \cdot f_{nq}(\mathbf{x}_q(t), \boldsymbol{\theta}_{nq}, \mathbf{m}_{nq}) \quad (4)$$

with the following two terms representing the interaction term f_{nq} of the parent $q \in \mathcal{P}_n$ with the gene n.

In case of an activating interaction between the parent gene $q \in \mathcal{P}_n$ and gene n:

$$f_n(\mathbf{x}_n(t), \boldsymbol{\theta}_n, \mathbf{m}_n) = \left(\frac{x_q(t)^{m_{nq}}}{\theta_{nq}^{m_{nq}} + x_q(t)^{m_{nq}}} \right) \tag{5}$$

In case of an repressing interaction between the parent gene $q \in \mathcal{P}_n$ and gene n:

$$f_n(\mathbf{x}_n(t), \boldsymbol{\theta}_n, \mathbf{m}_n) = \left(\frac{1}{1 + \left(\frac{x_q(t)}{\theta_{nq}} \right)^{m_{nq}}} \right) \tag{6}$$

Data simulated from the networks shown in figure 3 (main text) was simulated over the timespan $(0, 20)$ with a time-step of either 0.5 or 1.0 for deterministic data and 25 or 50 for stochastic data. Starting concentrations for the five genes $[x_1(0), \ldots, x_5(0)]$ were set as $[1.0, 0.5, 1.0, 0.5, 0.5]$ in all cases.

The following ODE systems were used for simulation of the oscillatory and non-oscillatory noise-free data:

Non-oscillatory data

$$\begin{aligned}
\dot{x}_1(t) &= 0.1 - 0.4 \cdot x_1(t) + 2.0 \cdot \frac{x_5(t)^2}{1.5^2 + x_5(t)^2} \\
\dot{x}_2(t) &= 0.2 - 0.4 \cdot x_2(t) + 1.5 \cdot \frac{x_1(t)^2}{1.5^2 + x_1(t)^2} \cdot \left(1 + \left(\frac{x_5(t)}{2} \right)^1 \right)^{-1} \\
\dot{x}_3(t) &= 0.2 - 0.4 \cdot x_3(t) + 2.0 \cdot \frac{x_1(t)^2}{1.5^2 + x_1(t)^2} \\
\dot{x}_4(t) &= 0.4 - 0.1 \cdot x_4(t) + 1.5 \cdot \frac{x_1(t)^2}{1.5^2 + x_1(t)^2} \cdot \left(1 + \left(\frac{x_3(t)}{1} \right)^2 \right)^{-1} \\
\dot{x}_5(t) &= 0.3 - 0.3 \cdot x_5(t) + 2.0 \cdot \frac{x_4(t)^2}{1.0^2 + x_4(t)^2} \cdot \left(1 + \left(\frac{x_2(t)}{0.5} \right)^3 \right)^{-1}
\end{aligned}$$

Oscillatory data

$$\dot{x}_1(t) = 0.2 - 0.9 \cdot x_1(t) + 2 \cdot \frac{x_5(t)^5}{1.5^5 + x_5(t)^5}$$
$$\dot{x}_2(t) = 0.2 - 0.9 \cdot x_2(t) + 2 \cdot \frac{x_1(t)^5}{1.5^5 + x_1(t)^5}$$
$$\dot{x}_3(t) = 0.2 - 0.7 \cdot x_3(t) + 2 \cdot \frac{x_1(t)^5}{1.5^5 + x_1(t)^5}$$
$$\dot{x}_4(t) = 0.2 - 1.5 \cdot x_4(t) + 2 \cdot \frac{x_1(t)^5}{1.5^5 + x_1(t)^5} + 2 \cdot \left(1 + \left(\frac{x_3(t)}{1.5}\right)^5\right)^{-1}$$
$$\dot{x}_5(t) = 0.2 - 1.5 \cdot x_5(t) + 2 \cdot \frac{x_4(t)^5}{1.5^5 + x_4(t)^5} + 2 \cdot \left(1 + \left(\frac{x_2(t)}{1.5}\right)^3\right)^{-1}$$

Raw Gene-Expression Time-Course Data Plots

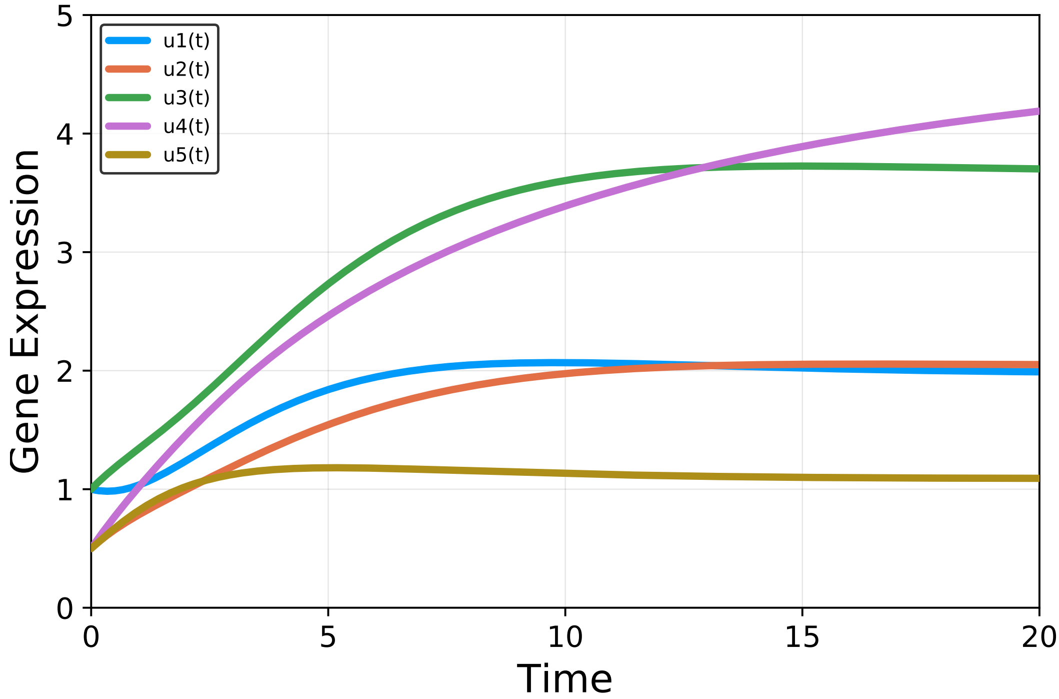

Figure 1: Solution to ODE system used for simulation of the non-oscillatory time-course data.

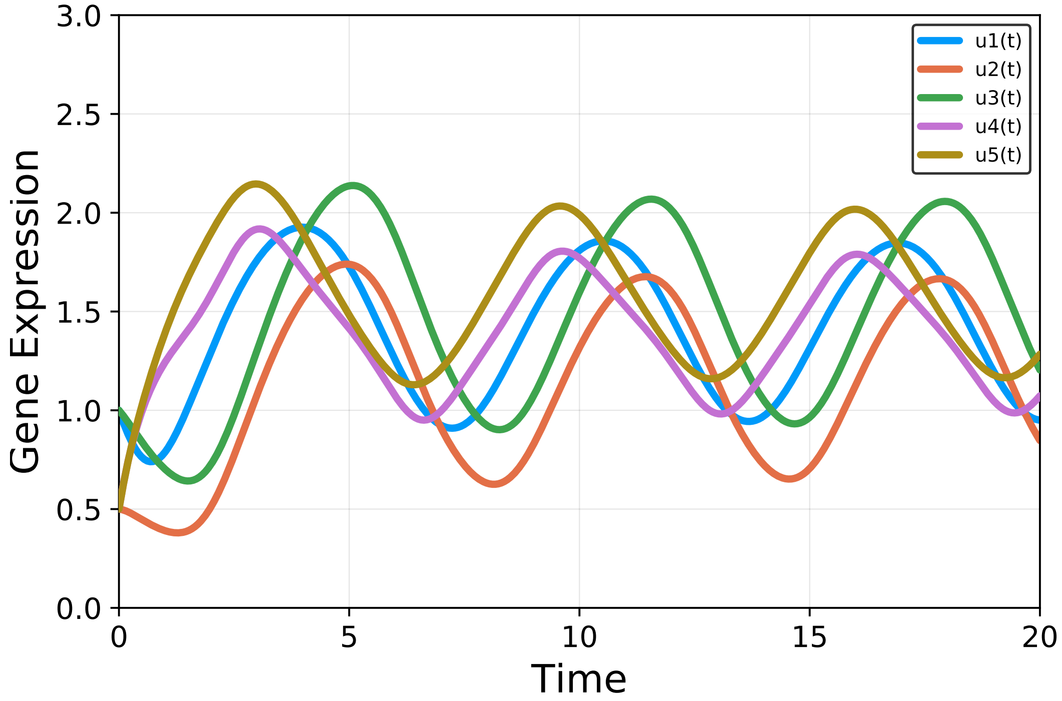

Figure 2: Solution to ODE system used for simulation of the oscillatory time-course data.

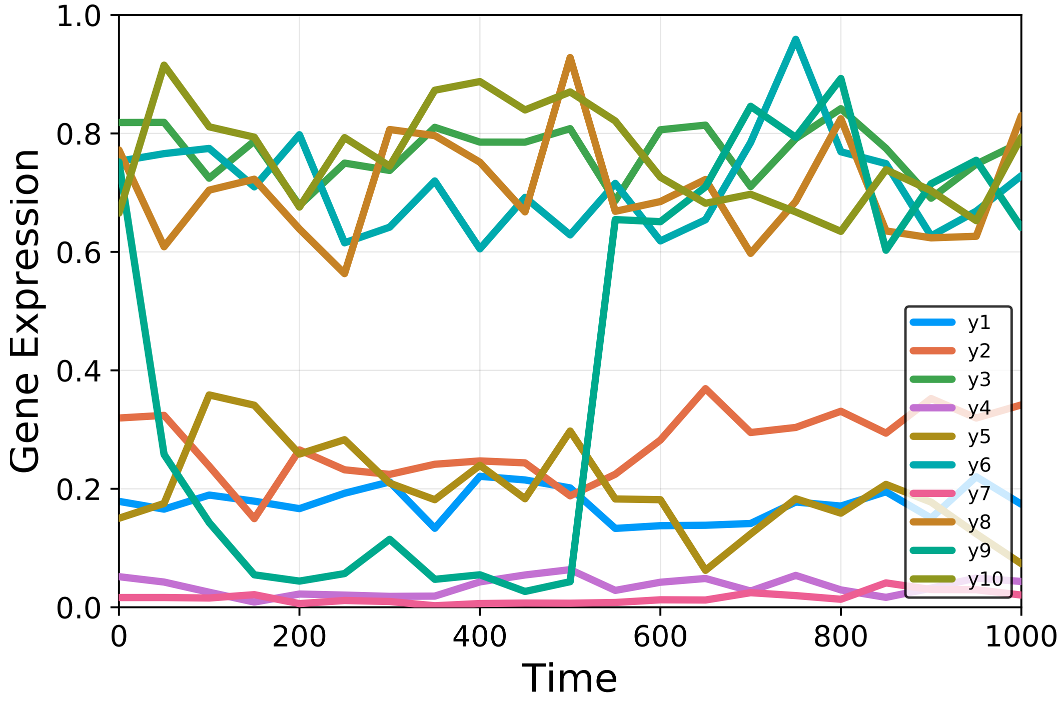

Figure 3: Realistic *in silico* time-course data simulated by `GeneNetWeaver`.

1.2 Parameters for Model Optimisation

Parameter optimisation of each putative decoupled model was done using either known bounds and starting values for each parameter or completely unconstrained optimisation. For constrained optimisation, basal transcription and degradation rates were assumed to be known, while they were included as free parameters for the unconstrained optimisation.

For the guidelines used to simulate the non-oscillatory data (equations 1, 2, 3 in Appendix 1.1 and figure 3A (main text)), this results in either $3 + 2 \cdot r$ or $1 + 2 \cdot r$ free parameters to be optimised for each decoupled gene, depending on whether basal transcription and degradation rates are known and where r is the number of parents for each gene. For the guidelines used to simulate the oscillatory data (equations 4, 5, 6 in Appendix 1.1 and figure 3B (main text)), the number of free parameters per decoupled ODE system is $2 + 3 \cdot r$ or $3 \cdot r$ respectively.

Parameter constraints and starting values for the remaining parameters optimised during constrained optimisation are displayed in Table 1. In case of unconstrained ODE optimisation, all free parameters were initialised at 1.0.

For non-parametric network inference, models were optimised using the likelihood (equation 3 (main text)) as the maximisation objective and starting values 1.0 for all free hyperparameters. Under certain circumstances (as mentioned in the main text) the length-scale hyperparameter of the GP was fixed at 50, 100, 150 or 200 before running the optimisation in order to achieve a satisfactory fit to the data.

Table 1: Constraints and and initial parameter values used for constrained optimisation of putative ODE models.

Data class	Param.	Initial value	Lower bound	Upper b.	True value(s)
'Stat. data'	β	1.0	0.0	5.0	2.0, 1.5
	m	2.0	0.1	5.0	1.0, 2.0, 3.0
	θ	1.0	0.0	4.0	0.5, 1.0, 1.5, 2.0
'Osc. data'	β	1.0	0.5	4.0	2.0
	m	1.0	0.7	5.0	5.0
	θ	1.0	0.2	3.0	1.5

1.3 Performance Evaluation using Precision-Recall curves

In this section, we explain and define how we evaluated performance of our inference method using precision-recall curves.

To obtain the AUPR, the vector of edge weights was first sorted. Each value in the vector was then iteratively chosen as the cut-off point between a positive and negative prediction (i.e. all larger weights are considered positive predictions and vice versa). It was therefore possible to calculate the precision and recall for each of these thresholds [**?**]. A graph was plotted using the obtained precision (y-axis) and recall (x-axis) values. The area under the curve arising from this plot is the AUPR (see figure 4).

The procedure for obtaining the AUROC is analogous, with the only difference that the false positive rate is used instead of the precision.

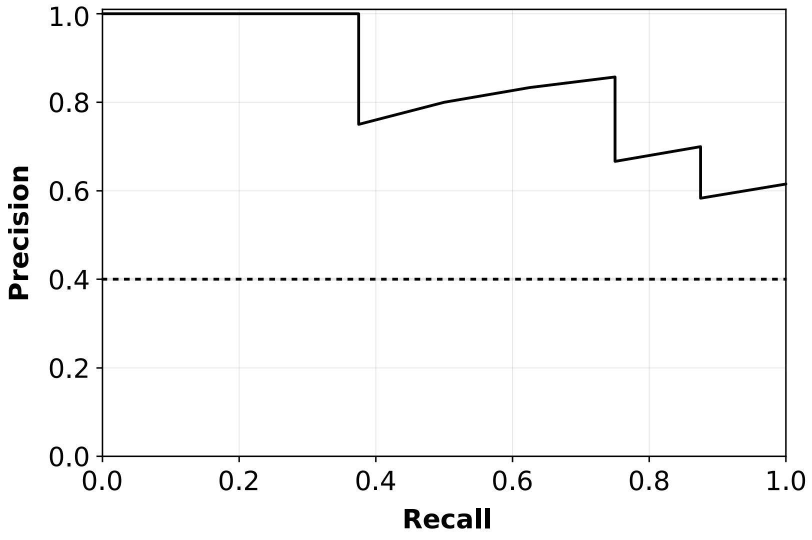

Figure 4: Example Precision-Recall curve. PR curve with AUPR of 0.84 with the baseline (random performance) shown as a dashed line.

Definitions

TP: True positive (a predicted edge which is actually present)

FP: False positive (a predicted edge which is not actually present)

TN: True Negative (an unpredicted edge which is actually not present)

FN: False Negative (an unpredicted edge which is actually present)

Precision (Positive Predictive Value) - the proportion of true edges that were predicted correctly:

$$\mathrm{PPV} = \frac{\mathrm{TP}}{\mathrm{TP} + \mathrm{FP}} \tag{7}$$

Recall (True Positive Rate) - the proportion of predicted edges that are actually present:

$$\mathrm{TPR} = \frac{\mathrm{TP}}{\mathrm{TP} + \mathrm{FN}} \tag{8}$$

False Positive Rate - the proportion of predicted edges that are not actually present:

$$\mathrm{FPR} = \frac{\mathrm{FP}}{\mathrm{FP} + \mathrm{TN}} \tag{9}$$

2 Supplementary Figures for Settings Evaluation

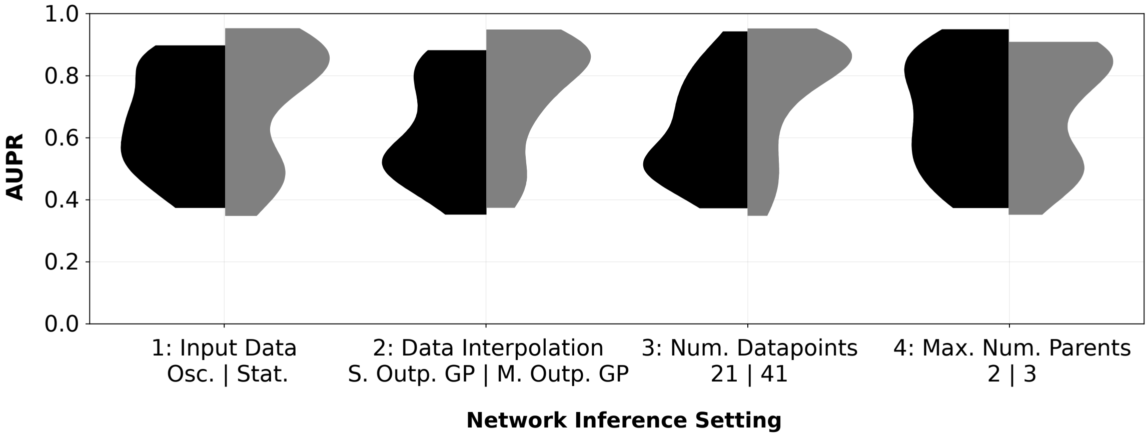

Figure 5: Performance of all evaluated inference approaches by model choices for noise-free data and inferring directed edges. This figure shows the impact of different settings choices on network inference performance. Summing the two halves of each of the four asymmetric distributions in the figure gives rise to the same distribution of model performance (constituted by distributions 1, 2 and 5 in figure 4 (main text)).

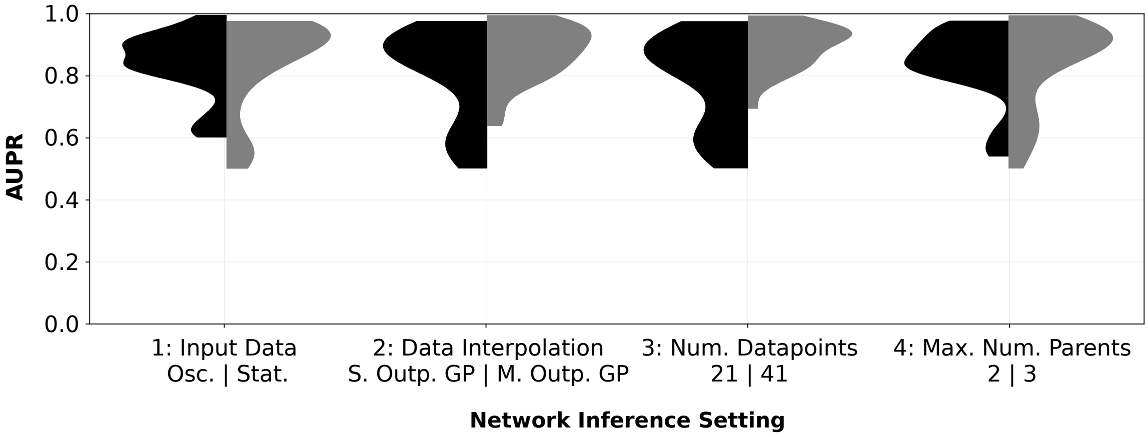

Figure 6: Performance of all evaluated inference approaches by model choices for noise-free data and inferring undirected edges. This figure shows the impact of different settings choices on network inference performance. Summing the two halves of each of the four asymmetric distributions in the figure gives rise to the same distribution of model performance (constituted by distributions 1, 2 and 5 in figure 4 (main text)).

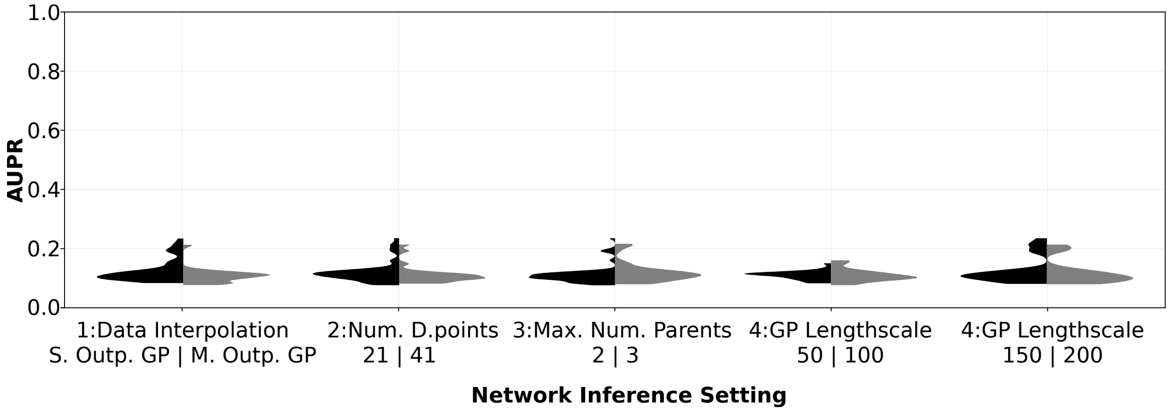

Figure 7: Performance of all evaluated inference approaches by model choices for stochastic data and inferring directed edges. This figure shows the impact of different settings choices on network inference performance. Summing the two halves of each of the first three asymmetric distributions in the figure gives rise to the same distribution of model performance (constituted by distributions 1 and 5 in figure 5 (main text)). The same is true for the sum of the last two distributions in the figure ("GP Lengthscale").

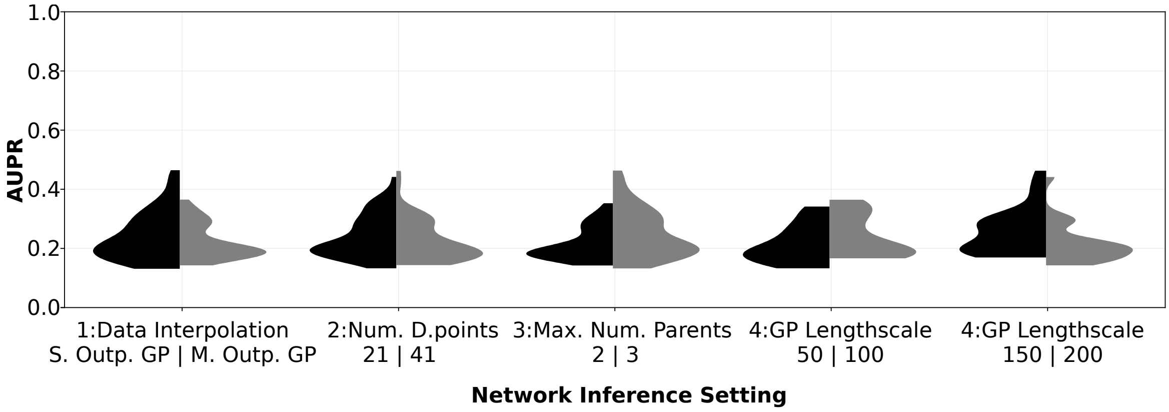

Figure 8: Performance of all evaluated inference approaches by model choices for stochastic data and inferring undirected edges. This figure shows the impact of different settings choices on network inference performance. Summing the two halves of each of the first three asymmetric distributions in the figure gives rise to the same distribution of model performance (constituted by distributions 1 and 5 in figure 5 (main text)). The same is true for the sum of the last two distributions in the figure ("GP Lengthscale").

3 Supplementary Figures for fitting GPs

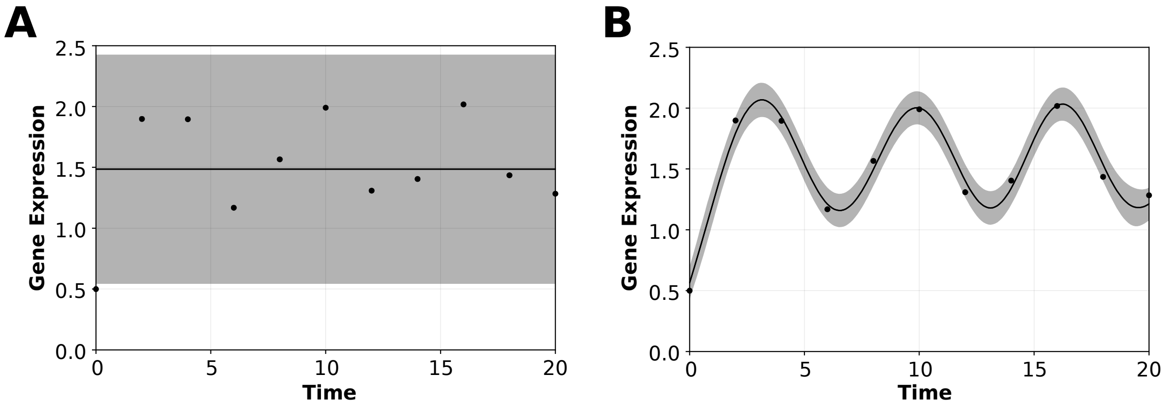

Figure 9: Comparison of single output GP and multiple output GP fits to data. Both plots show time-course data for the expression of gene 5 from the non-oscillatory (noise-free) data indicated by black dots. The black lines refer to the GP mean function with 95% confidence intervals shaded in grey. **A** Data interpolated using a single output GP. **B** Data interpolated using a multiple output GP, which takes into account correlations between the expression time-courses of all genes in the network.

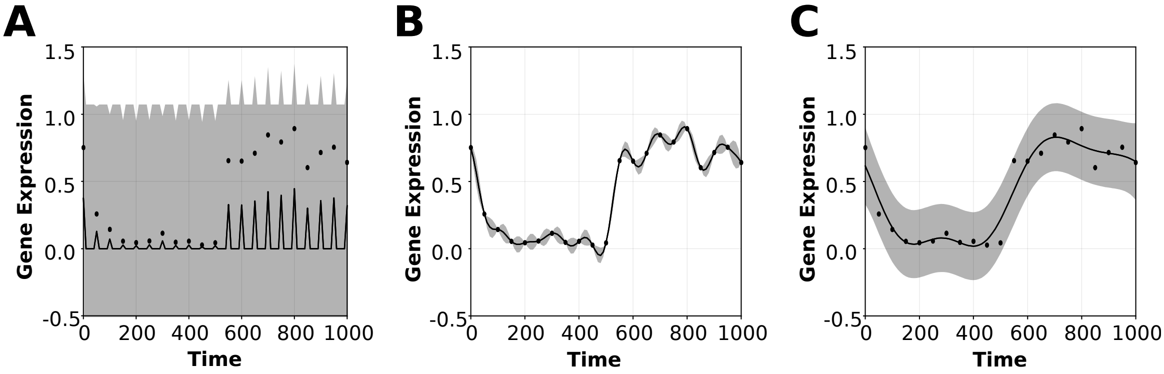

Figure 10: Comparison of GP fit with different length-scale hyperparameters. All three plots show the time-course data for the expression of gene 9 from the GNW dataset indicated by black dots. The black lines refer to the GP mean function with 95% confidence intervals shaded in grey. The three plots show different length-scale hyperparameter settings: **A** unconstrained hyperparameter optimisation, **B** length-scale fixed to 50, **C** length-scale fixed to 150.

4 Software Tools

All code for this project was written in the `Julia` programming language, version *0.6* [?].

4.1 Data Simulation

Simulations of the mRNA expression data described by the ODE systems shown is Section 1.1 were carried out using the Runge-Kutta Order 4 (`RK4()`) solver from `DifferentialEquations.jl`[1] [?].

4.2 Gaussian Process Regression

Gaussian Process regression was done using a squared exponential kernel (`RBF()`) the `GPy`[2] `Python` package called in `Julia` through `PyCall.jl`[3]. GP hyperparameters were optimised using the `optimize_restarts` attribute of the model with 3, 5 or 15 restarts. In certain cases (stochastic *in silico* gene expression data), the length-scale parameter of the RBF kernel was fixed prior to optimisation.

4.3 Parameter Optimisation

ODE parameter optimisation was done using the Nelder Mead [?] (`NelderMead()`) algorithm from `Optim.jl`[4]. In case of constrained parameter optimisation, this was carried out using the `Fminbox{NelderMead}()` method from the same package.

GP hyperparameter optimisation for the non-parametric inference approach was done using `GPy` as described in the previous section.

[1] `https://github.com/JuliaDiffEq`
[2] `https://github.com/SheffieldML/GPy`
[3] `https://github.com/JuliaPy/PyCall.jl`
[4] `https://github.com/JuliaNLSolvers/Optim.jl`
